# Arvid Carlsson, and the story of dopamine

**DOI:** 10.4103/0019-5545.58907

**Published:** 2010

**Authors:** Vikram K. Yeragani, Manuel Tancer, Pratap Chokka, Glen B. Baker

**Affiliations:** 1 Department of Psychiatry, Wayne State University, Detroit, MI, USA, India; 2 University of Alberta, Edmonton, Canada, India; 3 Acharya Nagarjuna University, AP, India

Arvid Carlsson was born in Uppsala, Sweden in 1923. Dr. Carlsson, a pharmacologist, is best known for his contributions on the neurotransmitter, dopamine, for which he won the Nobel Prize in 2000 for Medicine/Physiology. The co-recipients were Dr. Eric Kendel and Dr. Paul Greengard.

Dr. Carlsson entered Medical School in 1941 and his education was interrupted by several years of service in the Swedish armed forces. In 1951, he finished the M.L. degree, now equivalent to M.D. in North America. Later, he became a Professor at the University of Lund. In 1959, he moved to Goteborg University.

In 1957, Dr. Carlsson showed that dopamine was a neurotransmitter in the brain and not just a precursor of norepinephrine.[1] This was the prevailing view at that time. He also developed an assay to measure dopamine in the brain and found that the highest regional concentration existed in the basal ganglia. This finding led to his experiments on reserpine, which depleted dopamine and produced a loss of movement control. These symptoms were similar to the clinical symptoms seen in the neurological illness, Parkinsonism.[2,3] He did not end his investigations there, and showed that L-dopa, a precursor of dopamine, was effective treat symptoms of Parkinsonism. L-dopa is still one of the mainstays of drug treatment in Parkinsonism.

Dr. Carlsson was also instrumental in developing the ‘dopamine theory of schizophrenia’[4,5] and the role of dopamine in the development of extrapyramidal side-effects of antipsychotic medications. Inhibition of central dopamine function is a basic property common to many to antipsychotic drugs. The mesolimbic and nigrostriatal portions of the dopaminergic system are probably the main targets for the psychological and the extrapyramidal actions, respectively, of these drugs. The fact that dopaminergic hyperfunction induced by amphetamines or L-dopa may lead to a disturbance mimicking paranoid schizophrenia, further supporting the role of dopamine in mental function. Although a primary disturbance in dopamine function in schizophrenia cannot be ruled out, the intimate relationship between dopaminergic and other neuronal systems should be studied in more detail. The possible involvement of other amine, amino acid or peptide transmitters in schizophrenia cannot be disregarded. For example, there is now a large body of evidence supporting dysfunction of the glutamate receptors in schizophrenia.

Dr. Carlsson was also among the first researchers of the antidepressant compound, zimeldine, which was the first selective serotonin re-uptake inhibitor. The precursor of this drug was brompheniramine. Here, one should note that he did substantial work on the synthesis and metabolism of 5-hydroxytryptamine (serotonin) in the central nervous system.[6] However, zimeldine produced a serious neuro logical side-effect, Guillian-Barre syndrome, in a few patients and thus was withdrawn from the market. Thirteen cases of the Guillain-Barre syndrome were reviewed in an article in which the authors showed that all occurred with a similar relationship to treatment with zimeldine. The risk of developing Guillain-Barre syndrome was increased about 25-fold among patients receiving zimeldine, as compared with the natural incidence of the disorder. These cases substantiate strong evidence that Guillain-Barre syndrome may occur as a specific, probably immunologically mediated, complication of drug therapy.[7]

In the aging brain, there is a reduction of the levels of several transmitter substances and of the activities of enzymes involved in their synthesis and/or catabolism.[8] Carlsson stated that the sensitivity to the aging process varies for different transmitters and brain regions and that the dopamine neurons were more age-sensitive than most other neurons investigated by him. The metabolism of monoaminergic neurotransmitters is increased in the aging brain, as seen by increased metabolite/neurotransmitter ratios, and he suggested that this may compensate for the loss of the transmitter. In various types of dementia, including Alzheimer's disease (AD) and senile dementia of Alzheimer type (SDAT), there is a decrease in levels of several neurotransmitters as compared to age-matched controls. Recently observed changes in the lipid composition of the white matter, indicating demyelination, in the brains of patients with AD/SDAT, stress the importance of studying multifactorial aspects of dementia. Dr. Carlsson also emphasized that preventive measures may reduce the toxicity of oxygen and of autoxidation products in the brain.[9]

It is interesting to note that Dr. Carlsson opposed fluoridation of water as it is a violation of modern pharmacological principles, and he succeeded in his campaign along with hundreds of other scientists. It is also important to note that the incidence of dental caries was the same in Sweden as compared to fluoridated countries such as the USA. As British Columbia in Canada was considering fluoridation of water, Carlsson said, ‘I would advise against fluoridation. He reiterated that individual prophylaxis (treatment) is preferable on principle grounds and that it is equally effective’.[10]
